# Supplementary material for: A Novel Dual-Function Redox Modulator Relieves Oxidative Stress and Anti-Angiogenic Response in Placental Villus Explant Exposed to Hypoxia—Relevance for Preeclampsia Therapy
Source: Biology (Basel). 2023 Sep 12;12(9):1229. doi: 10.3390/biology12091229 (PMC10525611; doi:10.3390/biology12091229)
Supplement: Supplementary file 1 [file biology-12-01229-s001.zip › biology-2581266-supplementary.pdf]

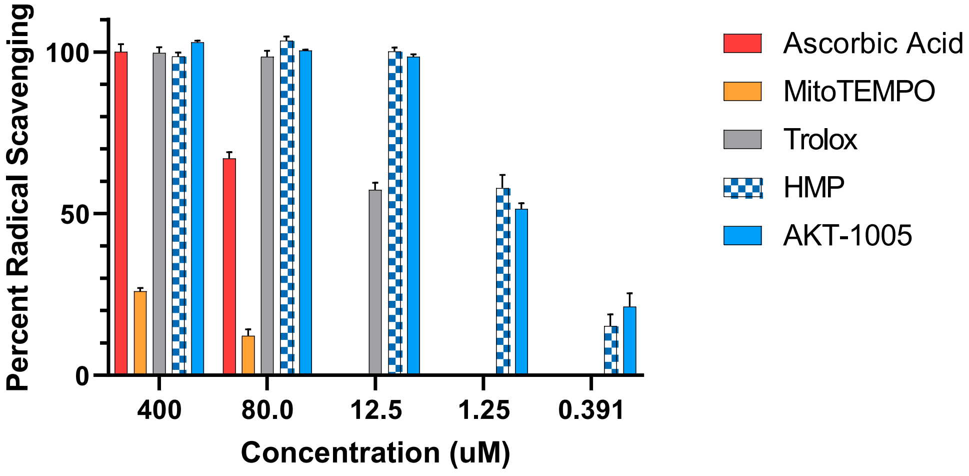

Supplemental Figure S1: Radical scavenging activity of AKT-1005 and HMP compared to the antioxidants Ascorbic Acid, MitoTEMPO, and Trolox at different concentrations in the Oxygen Radical Absorbance Capacity (ORAC) assay. (n = 3 per group).
